# Cutaneous Manifestations in Confirmed COVID-19 Patients: A Systematic Review

**DOI:** 10.3390/biology9120449

**Published:** 2020-12-05

**Authors:** Claudio Conforti, Caterina Dianzani, Marina Agozzino, Roberta Giuffrida, Giovanni Francesco Marangi, Nicola di Meo, Silviu-Horia Morariu, Paolo Persichetti, Francesco Segreto, Iris Zalaudek, Nicoleta Neagu

**Affiliations:** 1 Dermatology Clinic, Maggiore Hospital, University of Trieste, Piazza Ospitale 1, 34125 Trieste, Italy; claudioconforti@yahoo.com (C.C.); marinaagozzino@gmail.com (M.A.); nickdimeo@libero.it (N.d.M.); Iris.zalaudek@gmail.com (I.Z.); 2 Dermatology Section, Department of Plastic, Reconstructive and Cosmetic Surgery, Campus Biomedico University Hospital, Via Alvaro del Portillo 200, 00128 Rome, Italy; c.dianzani@unicampus.it; 3 Department of Clinical and Experimental Medicine, Dermatology, University of Messina, Piazza Pugliatti, 1, 98122 Messina, Italy; Roberta_giuffrida@hotmail.it; 4 Department of Plastic, Reconstructive and Cosmetic Surgery, Campus Biomedico University Hospital, Via Alvaro del Portillo 200, 00128 Rome, Italy; g.marangi@unicampus.it (G.F.M.); p.persichetti@unicampus.it (P.P.); f.segreto@unicampus.it (F.S.); 5 Dermatology Clinic, Mureș County Hospital, Nr. 12 Gheorghe Doja Street, 540015 Tîrgu Mureș, Romania; silviu_morariu@yahoo.com

**Keywords:** COVID-19, novel coronavirus, skin rash, cutaneous manifestations, histopathology, ocular

## Abstract

**Simple Summary:**

Patients diagnosed with COVID-19 and concomitant skin rashes have been frequently reported. We summarized the cases described to date, including only patients with positive RT-PCR testing from nasopharyngeal swabs. Six hundred and fifty-five patients were found who presented different types of skin rashes, from maculopapular, vascular, vesicular, urticarial, to atypical forms and ocular involvement. Chilblains which are lesions resembling frostbite have been more frequent in the younger population and seemed to predict a milder disease course. Vascular-purpuric lesions appeared in older patients and were linked to a more severe evolution. In the case of vesicular rashes, the possibility of herpesvirus co-infections was raised. Moreover, cutaneous hydroxychloroquine drug reactions have been described. For patients with conjunctivitis, eye discharge might be contagious. These skin manifestations may help identify asymptomatic COVID-19 carriers in some cases or predict a more severe evolution in others.

**Abstract:**

There have been increasing reports of skin manifestations in COVID-19 patients. We conducted a systematic review and included manuscripts describing patients with positive RT-PCR coronavirus testing from nasopharyngeal swabs who also developed cutaneous manifestations. A total of 655 patients were selected, with different types of skin rashes: Erythematous maculopapular (*n* = 250), vascular (*n* = 146), vesicular (*n* = 99), urticarial (*n* = 98), erythema multiforme/generalized pustular figurate erythema/Stevens-Johnson syndrome (*n* = 22), ocular/periocular (*n* = 14), polymorphic pattern (*n* = 9), generalized pruritus (*n* = 8), Kawasaki disease (*n* = 5), atypical erythema nodosum (*n* = 3), and atypical Sweet syndrome (*n* = 1). Chilblain-like lesions were more frequent in the younger population and were linked to a milder disease course, while fixed livedo racemosa and retiform purpura appeared in older patients and seemed to predict a more severe prognosis. For vesicular rashes, PCR determined the presence of herpesviruses in the vesicle fluid, which raised the possibility of herpesvirus co-infections. The erythema-multiforme-like pattern, generalized pustular figurate erythema and Stevens-Johnson syndrome were most frequently linked to hydroxychloroquine intake. A positive PCR determination of SARS-COV-2 from conjunctival swabs suggest that eye discharge can also be contagious. These cutaneous manifestations may aid in identifying otherwise asymptomatic COVID-19 carriers in some cases or predict a more severe evolution in others.

## 1. Introduction

Since December 2019, COVID-19 [1] has spread throughout the world at a staggering pace, gradually becoming a pandemic. As of September 19th, the confirmed number of cases has reached 30.827.639 globally and has claimed 958.514 lives [2]. The quantitative reverse transcriptase polymerase chain reaction (RT-PCR) is used to identify the viral nucleic acid in respiratory specimens or blood samples [3]. Common clinical features of COVID-19 include fever, cough, myalgia, fatigue, headache, and diarrhoea [4,5]. Although known to primarily affect the lungs and the respiratory function, recent reports from around the world have brought to our attention the possibility of cutaneous involvement [6]. These dermatologic symptoms may aid in identifying otherwise asymptomatic COVID-19 carriers or predict a more severe evolution in other cases. Therefore, we conducted a systematic review in order to collect clinically relevant information on the dermatologic effects of COVID-19.

## 2. Materials and Methods

A systematic review was elaborated following the Preferred Reporting Items for Systematic Reviews and Meta-Analyses (PRISMA) guidelines. A search of PubMed and Science.gov databases was performed for the period 2019–2020 using the terms: Coronavirus, COVID in combination with each of the following: Dermatology, skin, rash. Only articles in English were selected. The last search was run on 19 September 2020. Only manuscripts reporting on patients with positive RT-PCR-SARS-COV-2 testing from nasopharyngeal swabs who also developed cutaneous manifestations were included. Patients with underlying skin conditions were excluded. Eligible articles were assessed according to the Oxford Centre for Evidence-Based Medicine 2011 guidelines [7]. Review articles, meta-analyses, observational studies, case reports, survey snapshot studies, letters to the editor, and comments to the letters were all included. Other potentially relevant articles were identified by manually checking the references of the included literature.

Skin manifestations in patients with positive RT-PCR-SARS-COV-2 testing from nasopharyngeal swabs were assessed. The relation to COVID symptomatology and medication were analyzed. Histopathologic parameters, as well as RT-PCR testing from skin lesions and conjunctival swabs were summarized.

An independent extraction of articles was performed by two investigators according to the inclusion criteria. Disagreement was resolved by discussion between the two review authors. Since the study designs, participants, treatment measures, and reported outcomes varied markedly, we focused on describing the dermatologic findings, their relation to COVID-19 symptomatology, medication, histopathologic parameters, and other relevant investigations.

Limitations of this review lie in the form of confirmation bias in reporting, since every clinician interpreted these cases as either virus-related or medication-related. Moreover, epiphenomena cannot be excluded, as patients with skin rashes and flu-like symptoms might be increasingly likely to seek medical care, considering the actual pandemic situation. In order to limit bias in reporting COVID-19 related cutaneous manifestations, we only included patients with positive RT-PCR testing from nasopharyngeal swabs. Additionally, we objectively presented the skin manifestations as described by the initial authors and encompassed them into a table where mentions regarding the relation to systemic symptoms and medication intake were made. Moreover, histopathologic parameters, where available, were described for each type of skin rash. The interpretation of the initial authors was excluded. Skin manifestations were only categorized by morphology and not labelled as either virus-induced or medication-induced, thus leaving the final interpretation to the reader.

## 3. Results

**Figure 1:**
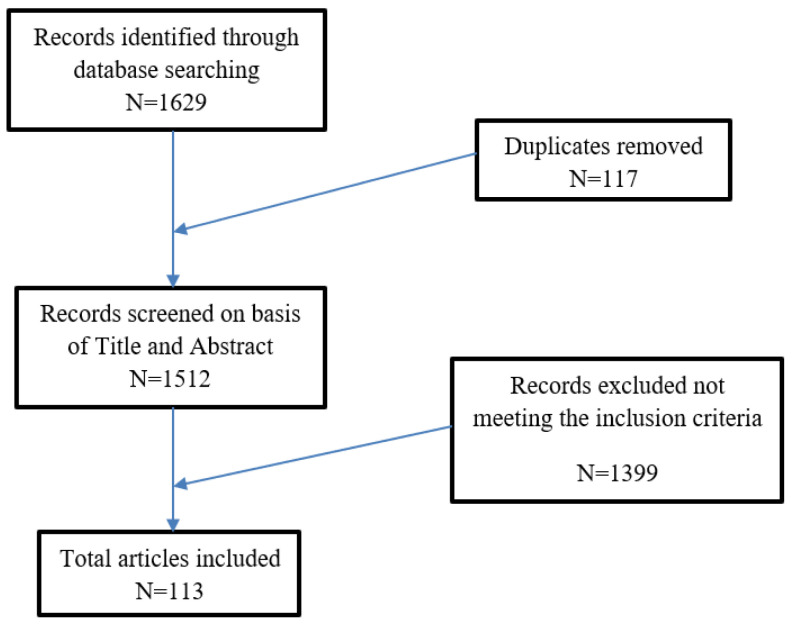
Literature search and article selection.

A total of 1629 records were initially identified in the literature search, of which 117 were duplicates. After screening for eligibility and inclusion criteria, 113 publications were ultimately included (Figure 1). The study and clinical characteristics are summarized in Table 1. The majority of publications were letters to the editor (*n* = 48), followed by case reports (*n* = 28), case series (*n* = 11), observational prospective studies (*n* = 4), and comments to letters (*n* = 2). All studies included were rated as level 4 or 5 evidence for clinical research as detailed in the Oxford Centre for Evidence-Based Medicine 2011 guidelines [7]. A total of 655 patients with dermatologic symptoms and positive RT-PCR-SARS-COV-2 testing from nasopharyngeal swabs were included.

Various cutaneous manifestations have been described. The most frequent were erythematous maculopapular (*n* = 250), followed by vascular lesions (*n* = 146), vesicular (*n* = 99), urticarial (*n* = 98), erythema multiforme/generalized pustular figurate erythema/Stevens-Johnson syndrome (*n* = 22), ocular/periocular (*n* = 14), polymorphic pattern (*n* = 9), generalized pruritus (*n* = 8), Kawasaki disease (*n* = 5), atypical erythema nodosum (*n* = 3), and atypical Sweet syndrome (*n* = 1).

### 3.1. Erythematous Maculopapular

Erythematous skin rashes (*n* = 250) have been the most frequent cutaneous manifestations in patients with RT-PCR confirmed COVID-19, with a majority of maculopapular patterns (*n* = 189) and macular erythema (*n* = 44), followed by papulo-squamous (*n* = 14) and one of each: Pityriasis rosea-like, Grover disease-like, SDRIFE-like [8,9,10,11,12,13,14,15,16,17,18,19,20,21,22,23,24,25,26,27,28,29,30,31,32,33,34,35,36]. The majority presented the rash onset after the appearance of COVID-19 symptoms (28 out of 33 cases that specified the timeframe).

Rosell-Diaz et al. conducted a retrospective case series on 12 adult patients with a mean age of 66 years (47–79 years). They had pneumonia and were on hydroxychloroquine, lopinavir/ritonavir treatment. After an average of 20.4 days (10–28) all patients developed papular exanthema, seven of which further developed erythema multiforme-like lesions and three of them presented fever and facial edema. Cutaneous biopsies were performed in two of these patients and were compatible with a drug reaction (Table 2). Underlying viral infections may increase the risk of adverse drug reactions, as it has already been described for the ampicillin rash in infectious mononucleosis or the increased risk of drug reactions in AIDS patients [8]. Antiviral immune responses may facilitate drug allergy and excessive production of proinflammatory cytokines, which has been observed in COVID-19 [101]. The authors strongly recommend that all COVID-19 patients with exanthema and eosinophilia be investigated for drug sensitization [8].

Sachdeva et al. reported three cases of COVID-19 with different cutaneous manifestations: A 72 year old female with a vesicular eruption, a 77 year old female with a morbilliform eruption on the trunk and legs, with purpuric areas on her legs and a 71 year old female with a maculopapular rash, which, interestingly, resembled Grover disease. In all three cases, the rash appeared after the onset of symptoms and two of them after a few days of treatment with HCQ, lopinavir/ritonavir, ceftriaxone [9].

Another peculiar skin rash was described by Estebanez et al.: A 28 year old female presenting with pruritic, confluent erythematous-yellowish papules on both heels, which appeared 10 days after the last dose of acetaminophen. No other skin lesions were present [5].

Tamai et al. described the onset of a maculopapular rash after 11–22 days from the initial symptoms of COVID-19, in three patients. One of them had six days of hydroxychloroquine and favipiravir treatment when the rash appeared. A drug induced eruption was excluded since erythema was relieved without discontinuing the medication [10].

A rash reminiscent of symmetrical drug-related intertriginous and flexural exanthema (SDRIFE) was reported by Mahé et al. It involved a 64 year old female who took acetaminophen 4 days previously. However, the rash disappeared after 5 days in spite of acetaminophen continuation, which excludes its involvement [11].

Sanchez et al. had an elderly patient who developed a digitate papulosquamous eruption clinically reminiscent of pityriasis rosea, 8 days after the onset of symptoms and 2 days after cefpodoxime discontinuation. He was admitted into the hospital after RT-PCR-SARS-COV-2 from nasopharyngeal swabs was positive. RT-PCR performed on a fresh skin biopsy specimen was negative for SARS-CoV-2 (Table 3). The PCR blood test for Epstein-Barr virus (EBV) was positive, with a viral load of 4.6 log10 copies/mL reflecting EBV replication. Serologic markers indicated reactivation and ruled out acute mononucleosis. The spontaneous resolution of the rash occurred within a week [12].

### 3.2. Vascular Lesions

Vascular lesions linked to COVID-19 were the second most frequently described in the literature [14,15,22,23,24,36,38,40,42,43,44,45,47,49,50,57,62,65,68,69,71,72,73,78,79,85,91,93,97,100,101,102,103,104]. Different patterns have been reported: Chilblain-like (*n* = 84), non-necrotic purpura (*n* = 5), necrotic purpura (*n* = 2), retiform purpura (*n* = 15), livedo reticularis (*n* = 23), livedo racemosa (*n* = 4), petechial rash (*n* = 4), eruptive cherry angiomas (*n* = 1), porcelain-like macules (*n* = 1), and dry gangrene (*n* = 7). The majority occurred after the onset of COVID-19 symptoms (25 out of 29 cases that specified the timeframe).

In a study by Freeman et al., chilblain-like lesions appeared in patients with relatively mild COVID-19 disease courses: Five out of thirty one hospitalized, two deaths [22]. They suggest that the underlying mechanism might be protective, making pernio (chilblain) a marker of a robust, effective host anti-viral response, limiting COVID-19 complications [36]. COVID-19 has been widely suspected as the etiological agent for these lesions, especially since they have been appearing in warm weather conditions. Clinical and histopathologic similarities to chilblain lupus erythematosus have been suggested. A type 1 interferon (IFN-I) mediated immune response is triggered in COVID-19 patients which plays an important part in the antiviral host defense, similar to the one in lupus erythematosus. It has been hypothesized that young patients exhibit an early IFN-I response, therefore muting early viral replication, but still inducing microangiopathic changes that cause a chilblain lupus erythematosus-like eruption. Older patients, however, may have an inadequate or delayed IFN-I response leading to an exacerbated hypercytokinemia with subsequent increased morbidity and mortality [68]. Galván et al. also linked the livedoid/necrotic lesions to older patients and severe disease (10% mortality) [15]. Fixed livedo racemosa, retiform purpura, and true acral ischemia appeared in critically ill patients [15,22,68]. Livedo racemosa and retiform purpura are hallmark manifestations of cutaneous thrombosis, appearing due to partial and complete occlusion of cutaneous blood vessels, respectively [51]. The appearance of livedo reticularis can be explained by the inflammatory effect of SARS-CoV-2 on endothelial cells or vessel-associated smooth muscle cells, both expressing angiotensin converting enzyme 2-receptor on their surface, which is the target of SARS-CoV-2-spike protein [97].

Droesch et al. described three cases of livedo racemosa and one case of retiform purpura. All four patients had markedly elevated D-dimer levels and complement including C5b-9 in skin biopsy samples [51]. Coagulopathy in the context of severe inflammation (elevated D-dimer, fibrinogen, or C-reactive protein levels) has been reported in patients with COVID-19 (Table 4) [102]. Elevated D-dimer is the most common laboratory abnormality and appears to be related to mortality [103]. The case reported by Bosch-Amate et al. highlights the concomitant presentation of cutaneous microthrombi presenting as retiform purpura and macrothrombi presenting as pulmonary thromboembolism in the setting of COVID-19 coagulopathy [44]. These morphologies are different from pernio-like lesions, as suggested by the histopathologic findings: Non-inflammatory to pauci-inflammatory thrombi without other findings linked to pernio, such as vacuolar interface changes, papillary dermal edema, and dermal lymphocytic infiltrate. This suggests that thrombotic disease in critically ill COVID-19 patients has a cutaneous correspondent manifesting in the forms of livedo racemosa, retiform purpura, or acro-ischemia. One study implicated activation of the alternative complement pathway in cutaneous thrombosis pathophysiology [22,72].

Almost 20% of COVID-19 patients present with severe disease consisting of microangiopathic ARDS and extrapulmonary thrombotic complications associated with markedly elevated D-dimers, which indicate an excessive activation of the coagulation pathway [72,104]. Magro et al. used pulmonary and cutaneous biopsy and autopsy samples from five patients with severe COVID-19 and demonstrated that critically ill cases are associated with generalized thrombotic injury. They appeared to be complement mediated: Extensive deposits of C4d, C5b-9, and MASP2 in the lungs of the two autopsied cases and C4d, C5b-9 in the three cases with a retiform and purpuric rash [72]. Similarly, Rotman et al. described a pauci-inflammatory thrombogenic vasculopathy with deposits of C3d, C4d, C5b-9, and MASP2 within the microvasculature and positive SARS-CoV-2 envelope/spike glycoprotein and ACE2 receptors in biopsy specimens [85].

Zhang Y et al. conducted a retrospective study on seven critically ill COVID-19 patients. They all had acral ischemia in the form of finger or toe cyanosis, skin bullae, and dry gangrene. D-dimer, fibrinogen, and fibrinogen degradation products were significantly elevated in most patients. Four patients were diagnosed with disseminated intravascular coagulation (DIC) and finally five patients died. The median time from acro-ischemia to death was 12 days [100].

### 3.3. Vesicular Rash

Vesicular rashes have been extensively reported [9,13,14,15,16,19,22,23,52,53,55,58,70,74,87,94,105,106]. We have gathered a total of 99 cases of RT-PCR confirmed patients who presented vesicular rashes. Specifically, three before, four concomitant with, and 52 after the onset of COVID-19 symptoms.

Fernandez-Nieto et al. described two different morphological patterns: A diffuse pattern, found in 18 patients (75%), which consisted of small papules, vesicles, and pustules of varying sizes, at different stages simultaneously, affecting more than one corporal area, including the palms and soles in two cases; a localized pattern found in six patients (25%), consisting of monomorphic lesions, at the same stage of evolution, with no more than one central area affected. Vesicular rashes appeared before COVID-19 symptoms in two cases, concomitant in three cases, and after in 19 cases with a median latency time of 14 days (range 4–30 days). An important mention is that seven patients had received HCQ, lopinavir/ritonavir, and azithromycin before the rash appeared. Multiplex PCR for herpesvirus and real-time RT-PCR for SARS-CoV-2 from the vesicle content performed in four cases were negative [55].

Llamas et al. also performed a herpesvirus family microarray PCR from the vesicle fluid in three patients with vesicular rash. The results showed a combination of HSV-1, HSV-6, and Epstein Barr virus (EBV) in case #1, HSV-1 and HSV-7 in case #2, and Varicella Zoster virus (VZV) in case #3. SARS COV-2 PCR in the vesicle fluid could not simultaneously be performed, which cannot completely rule out its additional involvement [70]. Vesicular exanthems are caused by viruses that can replicate in epidermal cells: DNA viruses, such as poxviruses, herpes simplex, varicella zoster [105], and some RNA viruses, including coxsackieviruses [106]. These findings bring into attention the possibility of a co-infection with herpesviruses, which might be responsible for the vesicular type of rash. However, the etiopathogenic role of SARS-COV-2 cannot be completely ruled out. Furthermore, reactivations of herpes simplex one HSV-1 [16] and herpes zoster have been reported in the evolution of COVID-19 [52,53,87].

### 3.4. Urticarial Rash

Urticarial rashes have been relatively frequent too. Ninety-nine cases have been described, either appearing before, concomitant with, or after COVID-19 symptoms, in similar proportions [13,14,15,16,17,18,19,21,22,24,39,41,48,54,56,59,60,61,82,89,96,99]. In four cases, drug intake could have played a part: Either acetaminophen [39], HCQ and azithromycin [56], HCQ, azithromycin, cefoperazone-sulbactam and omeprazole [59], or HCQ, lopinavir/ritonavir, ceftriaxon, enoxaparin [89]. In one of these cases, a skin biopsy was performed and the histopathologic image, along with a dermatological diagnosis of urticaria vasculitis led to an adverse drug reaction diagnosis [89]. However, in the case described by Gunawan et al., a patient developed facial urticaria after 3 days of hospitalization and HCQ, azithromycin, cefoperazone-sulbactam, and omeprazole treatment. Loratadine was added to his treatment with improvement on the next day. The suspicion of an adverse drug reaction was eliminated since the medication was not discontinued and still the rash improved [59].

Unique urticaria presentations include a case of annular urticaria plaques involving the upper limbs, chest, neck, abdomen of a 39 year old male, which appeared concomitantly with a 39 °C fever and no history of drug intake [41]. Moreover, a case of urticaria and angioedema of the face and hands in a 46 year old female appeared 2 days before the onset of COVID-19 symptoms and no history of drug intake, but with a known history of hay fever and mild asthma [60].

### 3.5. Erythema Multiforme/Generalized Pustular Figurate Erythema/Stevens-Johnson Syndrome

Twenty-two cases of erythema multiforme-like pattern have been described, of which three were generalized pustular figurate erythema and one Stevens-Johnson syndrome [8,20,24,37,46,63,75,84,86,107,108,109,110,111,112]. Sixteen of the patients described had a previous history of drug intake: Hydroxychloroquine, lopinavir/ritonavir, teicoplanin, azithromycin, acetaminophen, cefcapene, and loxoprofen, preceding the rash onset with 3 to 28 days [8,37,46,63,84,86]. A histopathological examination was performed in two of these cases and was compatible with a drug reaction [8].

Robustelli et al. described the case of a 70 year old woman presenting with a widespread eruption on an erythematous-oedematous base, with scattered pustules and scales, involving the face, trunk, upper limbs, and symmetric targetoid lesions over the buttocks, thighs, and legs. This eruption had a rapid onset and it appeared 3 days after treatment withdrawal: Lopinavir/ritonavir and hydroxychloroquine (HCQ) for 10 days for COVID-19 pneumonia. The patient had no personal or family history of psoriasis. A histopathological description was consistent with acute generalized exanthematous pustulosis (AGEP) [84].

AGEP is characterized by a sudden onset of widespread non-follicular sterile pustules arising within large areas of oedematous erythema and it has been secondary to drug intake in 90% of cases [107]. Rarely, atypical cases of AGEP with the development of target-like lesions have been described, especially in patients taking HCQ [108,109,110]. HCQ has been described as one of the main drugs involved in triggering AGEP [111,112]. AGEP has a rapid onset of up to 48 h of ingestion, often with an acute onset of fever and leukocytosis. Schwartz et al. brings into discussion another diagnostic entity, generalized pustular figurate erythema (GPFE), which has a longer onset, of 2 to 3 weeks after drug ingestion (range 4-27 days) and it is typically due to hydroxychloroquine. Previously described as atypical AGEP, GPFE may at first be evident as erythematous papules and plaques on the face, with facial edema and generalized urticaria, with development of nonfollicular pustules atop and finally erythematous and sometimes atypical targetoid erythema multiforme-like plaques on the trunk and extremities [107]. Taking into account the atypical clinical presentation and the longer onset of AGEP, the case reported by Robustelli et al. may fall into the GPFE category. Additionally, Abadias et al. described two cases of GPFE following 2 and 3 weeks, respectively, of HCQ treatment [37].

A retrospective case series by Rosell-Díaz et al. analyzed the appearance of a papular exanthema in 12 adult patients with RT-PCR diagnosed COVID-19 pneumonia. They all received hydroxychloroquine and lopinavir/ritonavir in combination with different other medications for 10 to 28 days before the rash onset. Seven of them developed target-like lesions and most of them had eosinophilia. A histopathological examination was performed in only two patients and was compatible with a drug reaction. The authors suggest that antiviral immune responses may induce drug sensitization via excessive production of proinflammatory cytokines, which has been observed in COVID-19. Moreover, exanthema and eosinophilia might be an indicator for an adverse drug reaction [8].

Erythema-multiforme (EM) pattern was reported in four cases by Rubio-Muniz et al., [24] one by Chaabane et al. [20], and one case of fever and rash at presentation, by Navaeifar et al., in a one year old male [75]. Jimenez-Cauhe et al. reported four cases of erythema-multiforme after 10-16 days of hydroxychloroquine, lopinavir/ritonavir, and azithromycin treatment [63]. Sakaida et al. described a case of EM pattern in a 52 year old female after 3 days of cefcapene and loxoprofen treatment for a dental procedure, 7 days after which she developed COVID-19 symptoms. In this case, drug eruption appeared in the latency period and it could be explained by a drug hypersensitivity expressed in some COVID-19 patients [86].

Davoodi et al. reported on a rare case of Stevens-Johnson syndrome in a 42 year old female with COVID-19 pneumonia which appeared after 2 days of treatment with HCQ and acetaminophen [46]. The rash resolved after HCQ discontinuation and lopinavir/ritonavir switch, thus demonstrating, at least in this case, that the cutaneous drug reaction was due to HCQ, and not lopinavir/ritonavir.

### 3.6. Ocular/Periocular Involvement

Ocular involvement has been described most frequently in the form of conjunctivitis (*n* = 11), but also as eyelid dermatitis and conjunctivitis (*n* = 1) and eyelid dermatitis (*n* = 2) [64,66,76,80,81,83,98,113].

Ping et al. evaluated 28 patients with positive RT-PCR testing from nasopharyngeal swabs. Of them, 11 presented ocular involvement in the form of conjunctival hyperemia, chemosis, epiphora, and increased secretions and two patients had positive findings for SARS-CoV-2 in their conjunctival swabs, as well [80]. Using the guideline on diagnosis and treatment of the novel coronavirus pneumonia issued by the National Health Commission of the People’s Republic of China, three cases were judged as moderate, two as severe, and six as critical [113], thus suggesting that ocular abnormalities occur in patients with more severe systemic manifestations.

Additionally, Kalner et al. reported two cases of recurrent dusky red, nonpruritic, nonblanching periorbital dyschromia in a 43 year old female and a 50 year old male, with moderate systemic symptoms. The dermatitis resolved with the resolution of systemic symptoms of COVID-19 [66]. Similarly, Olisova et al. reported the case of a 12 year old patient with periocular macular erythema with purpuric areas and strawberry tongue. The rash spontaneously resolved within 3 days [76].

Ocular involvement was reported in three other cases, one involving a 4 year old boy with polymorphic pattern: Bilateral nonpurulent conjunctivitis, strawberry tongue, erythematous lacy rash on the palms [98], and two cases with Kawasaki disease-like presentation [64,83].

### 3.7. Atypical Cutaneous Manifestations

Atypical COVID-related cutaneous manifestations include: Generalized pruritus with no skin lesions or previous drug history (*n* = 8) [21], atypical erythema nodosum (*n* = 3) [77,88,92], atypical Sweet syndrome (*n* = 1) [95], Kawasaki disease-like presentation (*n* = 5) [64,83,90], and polymorphic patterns (*n* = 9) [20,22,38,67,76,98,99].

Polymorphic patterns are unusual, especially when they appear in the same individual. A hypothesis to explain this polymorphism may be that in some cases there are alternative causes, which are different virus strains or different host reactions [15]. Aghazadeh et al. described the case of a 9 year old girl with vesicular oral eruption and acral erythematous papules and plaques [38]. Young et al. reported a 68 year old male with polymorphic pattern consisting of a morbilliform rash on his trunk, acral purpura and an ulcerated, purpuric plaque with livedoid borders on his buttocks [99]. The cases reported by Freeman et al. were a combination of either morbilliform and urticarial rash or morbilliform and pernio-like lesions [22]. Wolfe had a 4 year old patient who presented with bilateral nonpurulent conjunctivitis, strawberry tongue, and erythematous lacy rash on the palms [96]. Chaabane et al. described a pruriginous rash on the upper chest concomitant with unilateral livedo reticularis in a 35 year old woman [20]. Klimach et al. had a 13 year old patient with a maculopapular rash on his legs and chilblain-like lesions on the soles of his feet [67] and Olisova et al. a 12 year old patient with periocular macular erythema with purpuric areas and strawberry tongue [76]. Except for two cases [67,76], who had a few days history of acetaminophen intake, none of the others had any relation to a drug intake.

A noteworthy mention is the presence of cutaneous hyperesthesia [114]. In a letter to the editor, Krajewski et al. described the cases of two COVID-19 patients who developed abnormal hypersensitivity after the onset of fever and general symptoms, but which subsided 10 days after treatment inception [114].

## 4. Discussion

Erythematous maculopapular skin rashes were the most frequent cutaneous manifestation in COVID-19 patients. Cutaneous biopsies, although performed only in two of these patients, indicated a drug reaction [8]. However, the role of SARS-COV-2 cannot be completely excluded. It has been suggested that underlying viral infections may increase the risk of adverse drug reactions, as it was already established for the ampicillin rash in infectious mononucleosis or the increased risk of drug reactions in AIDS patients [8,101]. Patients with exanthema and eosinophilia might benefit from drug sensitization investigation.

Vascular lesions linked to COVID-19 were the second most frequently described in the literature. Chilblain-like lesions seem to be linked to a milder disease course and to affect the younger population [22,36,68]. Conversely, fixed livedo racemosa, retiform purpura, and true acral ischemia appeared in older, critically ill patients and seemed to predict a more severe prognosis [15,22,72]. This might be explained by an earlier IFN-I mediated immunologic response in young patients which mutes early viral replication. On the other hand, a delayed IFN-I response in older patients [68] along with coagulopathy [51,100,102,103] and alternative complement pathway activation [36,72,85,104] led to a generalized thrombotic state, clinically manifested as retiform purpura and pulmonary thromboembolism [44].

Vesicular rashes have been described as two different patterns, diffuse and localized. The majority appeared after the onset of COVID-19 symptoms and seven patients had received HCQ, lopinavir/ritonavir, and azithromycin before the rash appeared [55]. A herpesvirus family microarray PCR from the vesicle fluid was positive for different combinations of HSV-1, HSV-6, EBV, HSV-7, VZV in three patients. These findings raise the possibility of herpesvirus co-infection, which might be responsible for the vesicular type of rash [70], although the etiopathogenic role of SARS-COV-2 cannot be completely excluded. Multiplex PCR for herpesvirus and real-time RT-PCR for SARS-COV-2 from the vesicle content performed in four cases were negative [55]. Reactivations of HSV-1 [16] and herpes zoster [52,53,87] have also been reported in COVID-19 patients.

Urticarial eruptions have been described to appear either before or after COVID-19 symptoms or medication. Case reports have excluded drug reactions [59,60], as well as included them, with histopathological confirmation [39,55,59,89].

Erythema-multiforme-like (EM) pattern, generalized pustular figurate erythema (GPFE), and Stevens-Johnson syndrome (SJS) have been described in patients with COVID-19, the majority of whom had a history of drug intake [8,37,46,63,84,86]. A histopathological examination was performed in two of the cases and was compatible with a drug reaction [8]. Several articles in the literature have linked AGEP and GPFE to hydroxychloroquine (HCQ) [37,108,111,112]. Additionally, Davoodi et al. reported a SJS case after 2 days of HCQ and acetaminophen. The skin rash resolved, even though lopinavir/ritonavir was introduced instead of HCQ [46], which takes the focus off the lopinavir/ritonavir role, at least for this patient.

Ocular involvement in the form of conjunctivitis appears to be linked to a more severe form of COVID-19 disease. Ping et al. determined SARS-COV-2 in the conjunctival swabs of two patients, which suggests that eye discharge can also be contagious [80].

Atypical COVID-related cutaneous manifestations including generalized pruritus, atypical erythema nodosum, atypical Sweet syndrome, Kawasaki disease-like presentation, and polymorphic patterns have been reported [15,20,21,22,38,64,67,76,77,83,88,90,92,95,98,99]. Their etiopathogeny and relation to COVID-19 remain to be further investigated. A histopathological examination remains of paramount importance.

## 5. Conclusions

The high diversity of cutaneous manifestations linked to the novel coronavirus, coupled with the low availability of RT-PCR tests from nasopharyngeal swabs is still interfering with our ability to accurately classify each morphology as either COVID-19-related or drug-related. The histopathological examination, as well as RT-PCR testing from eye discharge and vesicular fluid bring us closer to an accurate diagnosis.

Alternative etiopathogenetic factors might be involved in the appearance of skin rashes in COVID-19 patients, such as herpesvirus co-infections or re-activations and drug reactions, especially hydroxychloroquine-related. The latter was proved to be more frequent in patients with underlying viral infections. However, vascular rashes, either IFN-I mediated or as a consequence of coagulopathy and alternative complement pathway activation, seem to be linked to the novel coronavirus. Furthermore, conjunctivitis appearing in COVID-19 patients might be highly contagious.

Given the actual pandemic restrictions, face-to-face consultations have been temporarily replaced by telemedicine. The possibility of photo and video sharing, as well as communication via text and voice messages have made the continuation of dermatologic examinations possible. Additionally, treatment adherence has been improved by telemedicine, as shown in a study conducted my Marasca et al. [115].

## Figures and Tables

**Table 1:** Study characteristics, patient demographics, and cutaneous symptoms in patients with COVID-19.

References	Level of Evidence	Country	Total Number of RT-PCR Confirmed Cases	RT-PCR Confirmed Cases with Cutaneous Involvement	Age (Years) And Sex (M/F)	Rash Type	Rash Location	Fever	Relation to Onset of COVID Symptoms	Relation to Medication
Abadias et al. [37]	4	Spain	2	2	M 64 F 60	GPFE	trunk, limbs, scalp, axillae	-	-	2 and 3 weeks after hydroxychloroquine, lopinavir/ritonavir; teicoplanin, azithromycin
Aghazadeh et al. [38]	5	Iran	1	1	F 9	polymorphic pattern: Vesicular oral eruption, and acral erythematous papules and plaques	oral; acral	yes	-	acetaminophen
Ahouach et al. [39]	5	France	1	1	F 57	urticaria	trunk, limbs	yes	2 days after	acetaminophen
Alramthan et al. [40]	4	Qattar	2	2	F 27, 35	chilblain-like	hands	-	-	-
Amatore et al. [41]	5	France	1	1	M 39	urticaria (annular)	upper limbs, chest, neck, abdomen	yes	concomitant	-
Andina et al. [42]	5	Spain	1	1	-	chilblain-like	acral	-	-	-
Annunziata et al. [19]	4	Italy	4	4	F 66 F 60 M 30 M 30	- macular - vesicular - vesicular - urticarial	- trunk - abdomen - trunk - legs	yes yes yes yes	2–10 days after	-
Avellana et al. [29]	5	Spain	1	1	F 32	maculopapular	generalized	yes	6 days after	after acetaminophen
Balestri et al. [43]	5	Italy	1	1	F 74	chilblain-like with digital infarcts and ischemic necrosis of the left third fingertip	acral	-	-	-
Bosch-Amate et al. [44]	5	Spain	1	1	F 79	retiform purpura	knee area	yes	-	-
Bouaziz et al. [14]	4	France	14	14	-	four macular erythema, two vesicular, one cold urticaria, seven vascular	-	-	a few days after	-
Chaabane et al. [20]	4	Tunisia	3	3	F 20 F 35 F 36	- erythema multiforme pattern - polymorphic pattern: Rash on upper chest and livedo reticularis on arm - maculopapular	- trunk, upper limbs, thighs -upper chest and arm - sub-mammary fold and trunk	yes no yes	3–4 days after	-
Conforti et al. [45]	5	Italy	1	1	F 62	transient livedo reticularis of the back, abdomen, and face; periorbital livedoid maculae	back, abdomen, and face	yes	14 days after	-
Dalal et al. [21]	4	India	102	13	Mean 39	three maculopapular, two urticaria, eight generalized pruritus	trunk and extremities	yes	2–3 days after	-
Davoodi et al. [46]	5	Iran	1	1	F 42	Stevens-Johnson syndrome with positive Nikolsky sign	generalized, orolabial and genital	yes	-	2 days after hydroxychloroquine, acetaminophen; then it was changed to lopinavir/ritonavir
de Masson et al. [47]	4	France	25	7	-	chilblain-like	acral	-	-	-
de Medeiros et al. [48]	5	Brazil	1	1	F 55	urticaria with palmar erythema	shoulders, inguinal region, palms	yes	concomitant	-
Diaz-Guimaraens et al. [49]	5	Spain	1	1	M 48	petechial	buttocks, popliteal fossae, proximal anterior thighs, and lower abdomen	yes	few days after	no
Dominguez-Santas et al. [50]	5	Spain	1	1	F 71	petechial	both legs, extending from the ankle and up to the thigh	yes	7 days after	no
Droesch et al. [51]	4	USA	4	4	-	three livedo racemosa one retiform purpura	hands and forearms	-	19–23 days after	-
Elsaie et al. [52]	5	Egypt	1	1	M 44	herpes zoster	upper chest and back	-	7 days after	-
Elsaie et al. [53]	5	Egypt	2	2	M 68 F 60	herpes zoster	- right thigh - chest and neck	yes no	- 2 days before - concomitant	-
Estebanez et al. [5]	5	Spain	1	1	F 28	maculopapular	heels	-	-	10 days after acetaminophen
Falkenhain-López et al. [54]	5	Spain	1	1	F 51	urticaria	trunk, thighs, upper limbs, face, dorsal aspects of hands	-	concomitant	no medication
Fernandez-Nieto et al. [55]	4	Spain	24	24	Median 45	vesicular	trunk, limbs, head, palms, soles	-	2 before, 3 concomitant, 19 after	In seven patients after hydroxychloroquine, lopinavir/ritonavir; azithromycin
Fernandez-Nieto et al. [56]	5	Italy	1	1	F 32	urticaria	lower trunk, thighs	-	6 days after	4 days after hydroxychloroquine and azithromycin
Freeman et al. [22]	4	31 countries	135	135	Median 45	32 morbiliform, 19 macular erythema, 14 papulo-squamous, 16 vesicular, 23 urticarial, 16 pernio, 10 retiform purpura, 5 polymorphic	-	-	-	-
Freeman et al. [36]	4	8 countries	14	14	Median 25	chilblain-like	acral	-	-	-
Galvan et al. [15]	4	Spain	375	234	-	29 chilblain-like, 17 vesicular, 49 urticarial, 122 maculopapular, 17 livedo/necrosis	-	-	22 before; 212 at the same time; 139 after	-
García-Legaz et al. [57]	4	Spain	2	2	-	chilblain-like papulosquamous	-	-	after	-
Genovese et al. [58]	5	Italy	1	1	F 8	vesicular	trunk	no	6 days after	-
Gianotti et al. [25]	4	Italy	3	3	F 59 F 89 M 57	maculopapular	- arms, trunk, and lower limbs - trunk and arms - generalized	yes yes yes	7 days after 2 days before	3 days after lopinavir-ritonavir, heparin and levofloxacin
Gianotti et al. [23]	4	Italy	8	8	-	four maculopapular, two vesicular, one of which presented with Grover disease-like pattern, two livedoid	-	-	-	-
Goncalves et al. [28]	5	Portugal	1	1	M 57	maculopapular	elbows and abdomen	yes	2 days after	-
Gunawan et al. [59]	5	Indonesia	1	1	M 51	urticaria	face	yes	5 days after	3 days after azithromycin, hydroxychloroquine, cefoperazone-sulbactam, omeprazole
Hassan et al. [60]	5	Scothland	1	1	F 46	urticaria with angioedema of the lips and hands	generalized; lips and face	no	2 days before	-
Hedou et al. [16]	4	France	103	5	Mean 47	2 macular erythema, 2 urticaria, 1 vesicular (HSV-1)	-	-	4 concomitant 1 (urticaria) before	-
Henry et al. [61]	5	France	1	1	F 27	urticaria	generalized	no	a few days before	-
Hunt et al. [30]	5	USA	1	1	M 20	maculopapular	generalized	yes	concomitant	-
Iancu et al. [33]	5	Romania	1	1	F 41	maculopapular	generalized	yes	17 days after	15 days after hydroxychloroquine, azithromycin, lopinavir/ritonavir
Jimenez-Cauhe et al. [62]	5	Spain	1	1	elderly M	purpuric	axilary	-	-	hydroxychloroquine, lopinavir/ritonavir
Jimenez-Cauhe et al. [63]	4	Spain	4	4	Mean 66	erythema multiforme pattern; enanthema	trunk, face, limbs; oral mucosa	-	16–24 days after	10-16 days after hydroxychloroquine; lopinavir/ ritonavir; azithromycin;
Jones et al. [64]	5	USA	1	1	F 6 months	Kawasaki disease	-	yes	1 day after	-
Joob et al. [65]	5	Thailand	1	1	-	petechial	-	yes	few days before	no
Kalner et al. [66]	4	USA	2	2	F 43 M 50	eyelid dermatitis	ocular	yes	2 days before	-
Klimach et al. [67]	5	UK	1	1	M 13	polymorphic pattern: Maculopapular, chilblain-like	axillary, plantar aspects of his feet	yes	-	after acetaminophen
Kolivras et al. [68]	5	Belgium	1	1	M 23	chilblain-like	acral	yes	3 days after	-
Landa et al. [69]	4	Spain	2	2	M 91 F 24	chilblain-like	acral	-	-	-
Llamas-Velasco et al. [70]	4	Spain	3	3	F 59 M 69 M 79	vesicular	trunk, perioral	yes	21–43 days after	-
Locatelli et al. [71]	5	Italy	1	1	M 16	chilblain-like	acral	-	3 days after	-
Macedo-Pérez et al. [34]	4	Mexico	1	1	M 33 M 36	macular erythema	trunk and limbs	yes yes	7 days after 3 days after	- -
Magro et al. [72]	4	USA	3	3	M 32 F 66 F 40	- retiform purpura - dusky purpuric patches - livedo racemosa	- buttocks - palms and soles - chest, legs and arms	yes yes yes	after	- hydroxychloroquine, azithromycin and remdesivir - hydroxychloroquine, enoxaparin -
Mahe et al. [11]	5	Italy	1	1	F 64	macular erythema	antecubital fossae, extended to the trunk and axillary folds	yes	4 days after	4 days after acetaminophen
Manalo et al. [73]	4	USA	-	2	M 67 F 47	unilateral livedo reticularis	-	-	after	-
Marzano et al. [74]	4	Italy	22	22	Mean 60	vesicular	trunk, limbs	yes	0–12 days after	-
Mizutani et al. [35]	5	Japan	1	1	M 69	macular erythema	abdomen and upper thighs	-	-	after 38 days of favipiravir, ampicillin, sulbactam, and ceftriaxone
Morey-Olivé et al. [18]	4	Spain	2	2	M 6 y F 2 months	- maculopapular - urticaria	- generalized, including palms - generalized, with sparing of palms and soles	yes yes	- 2 days after - 4 days before	-
Najarian et al. [31]	5	USA	1	1	M 58	maculopapular	generalized	yes	1 day after	-
Navaeifar et al. [75]	5	Iran	1	1	M 1	erythema multiforme pattern	generalized	yes	1 day after	-
Olisova et al. [76]	5	Russia	1	1	F 12	polymorphic pattern: Macular erythema with purpuric areas; strawberry tongue	upper eyelids, above the eyebrows, and in temporal region; tongue	yes	3 days after	after paracetamol
Ordieres-Ortega et al. [77]	5	Spain	1	1	F 57	atypical erythema nodosum	right leg	yes	-	8 days after hydroxychloroquine, lopinavir/ritonavir
Patel et al. [78]	5	UK	1	1	F 78	purpuric	back	yes	7 days before	-
Piccolo et al. [79]	4	Italy	2	2	12–16	chilblain-like	acral	-	-	-
Ping et al. [80]	4	China	28	11	-	conjunctivitis	ocular	-	-	-
Ping et al. [81]	5	China	1	1	2 y and 10 m	eyelid dermatitis and conjunctivitis	ocular	-	after	-
Quintana-Castanedo et al. [82]	5	Spain	1	1	M 61	urticaria	thighs, arms, forearms	yes	before	-
Recalati S et al. [13]	4	Italy	88	18	-	14 macular erythema; three urticarial; one vesicular	-		8 concomitant; 10 after	-
Riphagen et al. [83]	4	UK	2	2	M 14 M 6	Kawasaki Disease	-	yes yes	-	-
Rivera-Oyola et al. [17]	4	USA	2	2	M 60 F 60	- maculopapular - urticaria	generalized	yes yes	3 to 9 days after	-
Robustelli et al. [84]	5	Italy	1	1	F 70	atypical AGEP/ GPFE	generalized	-	-	3 days after treatment withdrawal; 13 days after treatment inception hydroxychloroquine, lopinavir/ritonavir
Rosell-Díaz et al. [8]	4	Spain	12	12	Mean 66	five maculopapular; seven erythema multiforme pattern	generalized	3/12	-	10-28 days after hydroxychloroquine, lopinavir/ritonavir
Rossi et al. [27]	5	Italy	1	1	M 34	maculopapular	generalized with sparing of palms and soles	yes	5 days after	a few days after acetaminophen
Rotman et al. [85]	5	USA	1	1	F 62	retiform purpura and calciphylaxis	bilateral lower extremities	-	21 days after	-
Rubio-Muniz et al. [24]	4	Spain	17	16	-	six maculopapular, two chilblain, four erythema multiforme pattern, two palpable purpura, two urticaria	-	-	-	-
Sachdeva et al. [9]	4	Italy	3	3	F 71 F 77 F 72	- maculopapular (Grover disease- like) - maculopapular with purpuric areas - vesicular	sub-mammary folds, trunk and hips	yes yes yes	- 4 days after	- a few days after - 1 day after lopinavir/ritonavir, hydroxychloroquine, ceftriaxone
Sakaida et al. [86]	5	Japan	1	1	F 52	erythema multiforme pattern;	trunk and limbs	yes	7 days before	3 days after cefcapene and loxoprofen
Sanchez et al. [12]	5	France	1	1	elderly M	pityriasis rosea-like	abdomen, trunk, thighs	yes	8 days after	after cefpodoxime
Shors et al. [87]	5	USA	1	1	F 49	herpes zoster	face	yes	7 days after	-
Sipfle et al. [88]	5	USA	1	1	F 54	atypical erythema nodosum	lower back, upper extremities, and face	-	-	-
Skorza et al. [89]	5	Italy	1	1	47	urticaria vasculitis	trunk	-	-	a few days after ceftriaxon, lopinavir/ ritonavir, hidroxicloroquine, enoxaparin
Spencer et al. [90]	4	USA	2	2	M 11 F 7	Kawasaki Disease	-	yes yes	5–7 days after	-
Suarez-Valle et al. [91]	4	Spain	3	3	-	Chilblain-like	acral	-	17 to 28 days after	-
Suter et al. [92]	5	Switzerland	1	1	M 42	atypical erythema nodosum	shins	yes	12 days after	-
Tahir et al. [93]	5	Dubai	1	1	M 47	targetoid rash with central necrosis; palpable purpura and areas of vesiculation; 1 cm tender ulcer of on the undersurface of the tongue, gingival and lingual purpura	extremities, buttocks, and lower trunk; oral mucosa	yes	concomitant	-
Tamai et al. [10]	4	Japan	3	3	M 54 M 24 F 81	maculopapular	-	yes yes yes	- 11 days after - 10 days after - 22 days after	- 6 days after hydroxychloroquine and favipiravir - -
Tammaro et al. [94]	4	Italy, Spain	-	3	-	vesicular	trunk	-	-	-
Taşkın et al. [95]	5	Turkey	1	1	F 61	atypical Sweet syndrome; aphthous ulcers	cheek, scalp, extremities, trunk; hard palate and oral mucosa	yes	-	-
Van Damme et al. [96]	5	Belgium	2	2	M 71 F 39	urticaria	generalized	yes	1–2 days before	-
Verheyden et al. [97]	5	Belgium	2	1	M 57	symmetric livedo reticularis	trunk, abdomen, and thighs	yes	after	-
Wolfe et al. [98]	5	USA	1	1	M 4	polymorphic pattern: Bilateral nonpurulent conjunctivitis, strawberry tongue, erythematous lacy rash on the palms	ocular, oral, palmar	yes	-	-
Young et al. [99]	5	USA	2	2	M 68 F 39	- polymorphic pattern: Morbilliform rash on his trunk, acral purpura and an ulcerated, purpuric plaque with livedoid borders on his buttocks - urticaria	- trunk, buttocks, acral - generalized	- yes	- - before	- -
Zengarini et al. [26]	5	Italy	1	1	F 67	macular erythema	neck, trunk, back, and proximal portions of upper and lower limbs	yes	concomitant with fever recurrence	after 1 month of hydroxychloroquine, omeprazole, piperacillin/ tazobactam, remdesivir
Zhang Y et al. [100]	4	China	7	7	median 59	vascular: Acro-ischemia with finger/toe cyanosis, skin bullae, and dry gangrene	acral	yes	-	2 and 3 weeks after

**Table 2:** Histopathological characteristics of cutaneous exanthema in COVID-19 patients.

References	Age Sex	Rash Type	Histopathology
Abadias et al. [37]	M 64 F 60	generalized pustular figurate erythema; drug eruption	Acanthotic epidermis with parakeratosis and numerous intracorneal, subcorneal, and intraepidermal pustules. Exocytosis of neutrophils and mild spongiosis were present at the periphery of the intraepidermal pustules.
Ahouach et al. [39]	F 57	diffuse fixed erythematous blanching maculopapular lesions	Slight spongiosis, basal cell vacuolation, and mild perivascular lymphocytic infiltrate.
Amatore et al. [41]	M 39	annular urticaria	Superficial perivascular infiltrate of lymphocytes without eosinophils, papillary dermal oedema, subtle epidermal spongiosis, mild lymphocyte exocytosis, lichenoid and vacuolar interface dermatitis with occasional dyskeratotic keratinocytes in the basal layer.
Bosch-Amate et al. [44]	F 79	painful retiform purpuric-violaceous rash	Multiple thrombi occluding most small sized vessels of the superficial and mid-dermis. Direct immunofluorescence showed the deposition of IgM, C3, and fibrinogen within superficial-to-deep dermal blood vessel walls.
de Masson et al. [47]		chilblain-like	Lichenoid dermatitis with a perivascular and eccrine mononuclear infiltrate and vascular microthrombi in two cases.
Diaz-Guimaraens et al. [49]	M 48	confluent erythematous macules and papules	Superficial perivascular lymphocytic infiltrate with abundant red cell extravasation and focal papillary oedema, along with focal parakeratosis and isolated dyskeratotic cells. No features of thrombotic vasculopathy were present.
Dominguez-Santas et al. [50]	F 71	purpuric macules and papules	Vessel damage with fibrinoid necrosis of vessel walls, transmural infiltration by neutrophils with karyorrhexis, leukocytoclasia, and extravasated erythrocyte, with granular deposition of C3 within vessel walls.
Droesch et al. [51]		livedo racemosa and retiform purpura	Pauci-inflammatory thrombogenic vasculopathy involving capillaries, venules, and/or arterioles or small arteries. In three of dermal arterial thrombosis was noted, reminiscent of antiphospholipid syndrome, without any diagnostic confirmation of these antibodies.
Fernandez-Nieto et al. [55]		varicella-like exanthem	Intraepidermal vesicles with mild acantholysis and ballooned keratinocytes.
Fernandez-Nieto et al. [56]	F 32	urticaria	Perivascular infiltrate of lymphocytes, some eosinophils, and upper dermal oedema.
Freeman et al. [36]		chilblain-like	Mild vacuolar interface dermatitis with dense superficial and deep lymphocytic inflammation, consistent with pernio versus connective tissue disease. No thrombi were noted.
Gianotti et al. [25]	F 59 F 89 M 57	erythematous macules and papules	Superficial perivascular dermatitis
Gianotti et al. [23]		four maculopapular rash, two chickenpox like, one of which presented with Grover disease-like pattern, two livedoid exanthemas	Papular phase: Exocytosis with minimal vacuolar changes near the dermal–epidermal junction. In one of these patients, there were nests of intraepidermal Langerhans cells associated with signs of vasculitis and extravasation of red blood cells. Chickenpox eruption: Characteristic clefts in the lower epidermis, as well as dyskeratotic keratinocytes in the granular layer and also near the basement membrane. Livedoid exanthematous eruption: Nest of Langerhans cells in the epidermis. In the deep dermis and occasionally in the superficial dermis, there were microthrombi admixed with nuclear and eosinophilic debris.
Jimenez-Cauhe et al. [63]	F 58-77	erythema multiforme-like eruption	Normal basket-weave stratum corneum, and mild to moderate spongiosis in epidermis. The dermis showed dilated vessels filled with neutrophils, extravasation of red blood cells, and lymphocytic perivascular and interstitial infiltrate. Basal vacuolar changes with interface dermatitis were observed in one patient, and lymphocytic exocytosis in another.
Kolivras et al. [68]	M 23	chilblain-like	Superficial and deep lichenoid, perivascular, and perieccrine infiltrate of lymphocytes, with occasional plasma cells; necrotic (apoptotic) keratinocytes.
Locatelli et al. [71]	M 16	chilblain-like	Oedema of the papillary dermis, superficial and deep lymphocytic infiltrate in a perivascular and strong perieccrine pattern; there were no signs of endothelial damage.
Macedo-Pérez et al. [34]	M 33 M 36	maculopapular rash	Nonspecific mild to moderate dermatitis with isolated areas with interphase dermatitis and isolated apoptotic bodies.
Magro et al. [72]	M 32 F 66 F 40	- retiform purpura - dusky purpuric patches - livedo racemosa	Striking thrombogenic vasculopathy accompanied by extensive necrosis of the epidermis and adnexal structures, including the eccrine coil. There was a significant degree of interstitial and perivascular neutrophilia with prominent leukocytoclasia. IHC showed striking and extensive deposition of C5b-9 within the microvasculature. Superficial vascular ectasia and an occlusive arterial thrombus within the deeper reticular dermis in the absence of inflammation. Extensive vascular deposits of C5b-9, C3d, and C4d were observed throughout the dermis, with marked deposition in an occluded artery. A biopsy of normal-appearing deltoid skin also showed conspicuous microvascular deposits of C5b-9. Modest perivascular lymphocytic infiltrate in the superficial dermis along with deeper seated small thrombi within rare venules of the deep dermis, in the absence of a clear vasculitis. Significant vascular deposits of C5b-9 and C4d.
Rivera-Oyola et al. [17]	M 60 F 60	maculopapular rash urticaria	Mild perivascular infiltrate of predominantly mononuclear cells surrounding the superficial blood vessels and epidermis showed scattered foci of hydropic changes along with minimal acanthosis, slight spongiosis, and foci of parakeratosis.
Robustelli et al. [84]	F 70	AGEP; drug eruption	Subcorneal pustule with mild focal acanthosis and spongiosis, neutrophilic exocytosis, sparse keratinocyte necrosis, and a perivascular lymphocytic infiltrate with rare neutrophils and eosinophils, consistent with AGEP.
Rosell-Díaz et al. [8]		papular exanthema; seven patients developed target-like areas; three developed fever and facial edema;	One of them showed a superficial perivascular inflammation with eosinophils and the other showed a lichenoid pattern with eosinophils.
Rotman et al. [85]	F 62	retiform purpura plaques with concomitant calciphylaxis	Occlusive luminal thrombi and focal mural fibrin deposition.
Rubio-Muniz et al. [24]		six maculopapular rash, two chilblain, four targetoid lesions, two palpable purpura, two urticaria	Maculopapular: In early-onset cases, histopathology showed moderate epidermal spongiosis and perivascular lymphocytic infiltrate with eosinophils in the dermis, whereas the analysis of the delayed lesions showed perivascular lymphocytic infiltrate and histiocytes among collagen fibers without mucin deposits.
Sakaida et al. [86]	F 52	erythema multiforme-like; drug eruption	Interface changes with liquefaction and perivascular mixed cell infiltrations in the papillary dermis are observed. There are histiocytic infiltrations around the capillary vessels and neutrophils are scattered in the upper dermis.
Sanchez et al. [12]	elderly M	digitate papulosquamous eruption	Foci of spongiosis with focal parakeratosis in the epidermis and a few rounded spongiotic vesicles containing aggregates of lymphocytes and Langerhans cells. A moderate lymphohistiocytic infiltrate was present in the superficial dermis and was associated with papillary dermal oedema.
Skorza et al. [89]	47	urticarial vasculitis; drug eruption on EHP	Orthokeratotic hyperkeratosis, spongiosis, focal vacuolar degeneration of basal keratinocytes, and focal lymphocytic exocytosis. Slight inflammatory lymphomorphonuclear infiltrate of superficial dermis with minimal perivascular neutrophilic component was observed, with occasional aspects of vessel wall damage.
Suarez-Valle et al. [91]		Chilblain-like	Ischemic necrosis affecting the epidermis and dermis with signs of re-epithelialization. Vasculitis or microthrombi were not found after reviewing extensive deep sections.
Tahir et al. [93]	M 47	targetoid rash with central necrosis; palpable purpura and areas of vesiculation; 1 cm tender ulcer of on the undersurface of the tongue, gingival, and lingual purpura	Endothelial swelling, neutrophilic vessel wall infiltration, karyorrhectic debris, and fibrin deposition in small and medium-sized dermal vessels with extravasated erythrocytes. There were microthrombi occluding lumina of smaller dermal capillaries.
Taşkın et al. [95]	F 61	atypical Sweet syndrome with aphthous ulcers	Diffuse neutrophilic infiltration in the upper dermis and vascular proliferation with swollen endothelial cells and extravasated erythrocytes.
Young et al. [99]	M 68 F 39	- polymorphic pattern: Morbilliform rash on his trunk, acral purpura reminiscent of perniosis, and an ulcerated, purpuric plaque with livedoid borders on his buttocks - urticaria	Groups of apoptotic keratinocytes in the epidermis, suggestive of a viral exanthema. A biopsy from the buttocks showed features consistent with thrombotic vasculopathy.
Zengarini et al. [26]	F 67	erythematous confluent rash	Haematoxylin–eosin-stained tissue specimens showed slight superficial perivascular lymphocytic infiltrate, extremely dilated vessel in the papillary and mid dermis.

**Table 3:** Polymerase chain reaction (PCR) determinations from skin lesions in COVID-19 patients.

References	Cutaneous Manifestation	PCR Skin Lesions
Ahouach et al. [39]	maculopapular rash	negative
Dominguez-Santas et al. [50]	maculopapular rash	negative
Fernandez-Nieto et al. [55]	vesicular rash	negative four tested
Llamas-Velasco et al. [70]	vesicular rash	PCR in the vesicle fluid: A combination of Herpes Simplex-1 virus, Herpes Simplex-6 virus, and Epstein Barr virus in case #1, Herpes Simplex-1 virus and Herpes Simplex-7 in case #2, and Varicella Zoster virus in case #3.
Ping et al. [80]	conjunctivitis	two positive from conjunctival swabs
Sanchez et al. [12]	digitate papulosquamous eruption	negative

**Table 4:** Modifications of cutaneous markers in COVID-19 patients.

References	Cutaneous Manifestation	Coagulation Markers
Bosch-Amate et al. [44]	painful retiform purpuric-violaceous patches of 15 cm with some hemorrhagic blisters and crusts on both legs	D-Dimer of >10,000 ng/mL (reference value, <500).
Droesch et al. [51]	livedo racemosa on hands and forearms in three patients and retiform purpura on hands and forearms	All four patients had D-dimer levels of more than 3 μg/mL (normal range, 0-0.229 μg/mL)
Magro et al. [72]	retiform purpura, buttocks	elevated D-dimer of 1024 ng/mL (normal range 0–229) on presentation, which peaked at 2090 ng/mL on hospital day 19, and a persistently elevated INR of 1.6–1.9, but a normal PTT and platelet count.
	dusky purpuric patches, palms, and soles	markedly elevated D-dimer of 7030 ng/mL, but normal INR and PTT
	reticulated eruptions, consistent with livedo racemosa, chest, legs, and arms	D-dimer was elevated at 1187 ng/mL, with a normal platelet count and PTT, but an elevated INR of 1.4.
Suarez-Valle et al. [91]	chilblain-like eruption	D-dimer was elevated in the three of them and fibrinogen in two, but no other coagulation abnormalities were detected
Verheyden et al. [97]	symmetric livedo reticularis	elevated D-dimers
